# Anti-Inflammatory Therapeutic Mechanisms of Natural Products: Insight from Rosemary Diterpenes, Carnosic Acid and Carnosol

**DOI:** 10.3390/biomedicines11020545

**Published:** 2023-02-13

**Authors:** Solomon Habtemariam

**Affiliations:** Pharmacognosy Research & Herbal Analysis Services UK, University of Greenwich, Central Avenue, Chatham-Maritime, Kent ME4 4TB, UK; s.habtemariam@herbalanalysis.co.uk; Tel.: +44-208-331-8302 (ext. 8424)

**Keywords:** rosemary, diterpenes, carnosic acid, carnosol, inflammation, MAPK, NLRP3 inflammasome, Nrf2, NF-κB, SIRT1, STAT3

## Abstract

Carnosic acid (CA) and carnosol (CAR) are two major diterpenes of the rosemary plant (*Rosmarinus officinalis*). They possess a phenolic structural moiety and are endowed with the power to remove cellular reactive oxygen species (ROS) either through direct scavenging reaction or indirectly through upregulation of antioxidant defences. Hand in hand with these activities are their multiple biological effects and therapeutic potential orchestrated through modulating various signalling pathways of inflammation, including the NF-κB, MAPK, Nrf2, SIRT1, STAT3 and NLRP3 inflammasomes, among others. Consequently, they ameliorate the expression of pro-inflammatory cytokines (e.g., TNF-α, IL-1 and IL-6), adhesion molecules, chemokines and prostaglandins. These anti-inflammatory mechanisms of action as a therapeutic link to various effects of these compounds, as in many other natural products, are scrutinised.

## 1. Introduction

Any immune response to tissue insult resulting from infection or physical/chemical injury involves an initial stage of pro-inflammatory activity followed by a period of resolution of inflammation called the anti-inflammatory phase. The regulated process of inflammation allows the body to clear pathogens, dead cells and debris, by which means a normal tissue structure and homeostasis are sustained. Acute inflammation is largely a function of resident immune cells (dendritic cells, macrophages, mast cells, etc.) and is characterised by a temporal and limited upregulation of inflammatory activity followed by prompt resolution once the insult stimulus is removed. The classic example of an acute inflammatory response is that initiated through infection by pathogens. The process involves the interaction of pathogen-associated molecular patterns (PAMPs) expressed on pathogens with host immune cells’ receptors called pattern recognition receptors (PRRs) or danger-associated molecular pattern (DAMP) receptors. The PPRs include the Toll-like receptors (TLRs) as well as nucleotide-binding oligomerisation domain (NOD)-like receptors (NLR) (and mannose-binding lectin (MBL)) [1,2,3], which are all activated by various extracellular bacterial (e.g., endotoxins) and viral products [4] as well as cellular molecules (e.g., uric acid, ATP and DNA) released from damaged cells [5]. We also have non-TLRs as PRRs that activate the innate immune complexes, the inflammasome system [6,7]. Any physical, chemical or inflammatory signal of metabolic origin can also initiate an acute inflammatory response due to the release of DAMPs [8]. Meanwhile, chronic inflammatory conditions constitute long-term inflammatory activity that involves continuous recruitment and infiltration of leucocytes at the site of injury. This results in major structural and physiological alterations in the tissues and organs involved, resulting in diseases such as arthritis, asthma, type 2 diabetes (T2D) and autoimmune, cardiovascular and neurodegenerative (e.g., Alzheimer’s and Parkinson’s) diseases. The most prominent mediators of chronic inflammation are pro-inflammatory cytokines such as interleukin (IL)-1α, IL-1α, IL-1β, IL-6 and tumour necrosis factor-α (TNF-α). Not surprisingly, the most promising current therapeutic strategies for treating chronic inflammatory conditions are based on antibodies targeting pro-inflammatory cytokines (e.g., [9,10]) such as TNF-α (infliximab, adalimumab, etanercept, golimumab and certolizumab), IL-6 (targeting its receptor with tocilizumab or sarilumab or targeting the cytokine with siltuximab) and IL-1 (targeting its receptor with anakinra or rilonacept by binding with IL-1α and IL-1β, and canakinumab as an anti-IL-1β antibody). Other mediators include lipids of cyclooxygenase-2 (COX-2) products such as prostaglandin-E_2_ (PGE_2_), chemokines (proteins, lipids and other origins) and nitric oxide (NO) as a product of the inducible NO synthase (iNOS), among others. Antagonists of lipid mediators among the old-generation aspirin-like drugs (nonselective COX inhibitors) and the latest selective inhibitors (COX-2 inhibitors), as well as lipoxygenase inhibitors, are common anti-inflammatory agents on the market, though they are mostly of limited use for chronic inflammatory diseases. Following the release of inflammatory mediators, adhesion molecules both on leucocytes and endothelial cell surfaces are activated/upregulated, resulting in extravascular immigration of leucocytes, which also involves leucocytes’ interaction with extracellular matrix (ECM) proteins. Given the crucial role of leucocyte adhesion in the pathology of chronic inflammatory diseases, therapeutic strategies using antibodies are also common (e.g., natalizumab against human α_4_ integrin; see also the review by Slack et al. [11]). Linking the expression of inflammatory mediators to activation of PRRs are drug targets of the signal transduction pathways such as the mitogen-activated protein kinase (MAPK) [12] and transcription factors [13], including the nuclear factor ‘kappa-light-chain-enhancer’ of activated B-cells (NF-κB). All these pro-inflammatory pathways, as well as those of inflammation resolution, such as that by IL-10 production [14], are subject to modulation by anti-inflammatory agents including natural products. In the following sections, the therapeutic potential of the two most abundant rosemary diterpenes, carnosic acid (CA) and carnosol (CAR), orchestrated through multiple anti-inflammatory mechanisms is discussed. 

## 2. Phytochemical Overview

**Figure 1:**
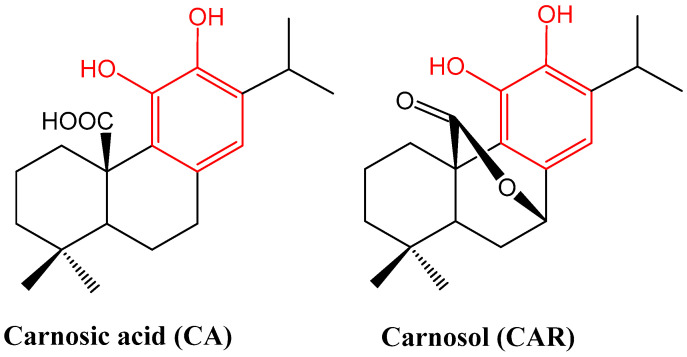
Structures of carnosic acid and carnosol. One common structural moiety for the two compounds is the orthodihydroxybenzene (catechol) group shown in red. This makes them similar to polyphenolic compounds such as flavonoids, which are known for their general antioxidant properties. They are also made from the 20-carbon diterpene skeleton. The concentration of CA can be up to 5-fold higher than CAR, but oxidation under various conditions can transform it into other products including CAR [17].

The chemistry and biosynthesis route of carnosic acid and carnosol (Figure 1) have been outlined previously [15]. They belong to the labdane type of diterpenes of the 20-carbon skeleton. What is unique about these compounds is that they possess a phenolic structural moiety which is common in flavonoids and other aromatic compounds of the shikimic acid and acetate metabolic pathways in plants. Hence, they are commonly known for their antioxidant properties as they can directly interact with reactive oxygen species (ROS). These two compounds are the major constituents of the aerial parts of rosemary, which also contain other related diterpenes including glycoside forms at minor concentrations [15]. They have a very limited distribution in plants and have been isolated from few genera of the Lamiaceae family (including *Salvia* (sage such as *S. officinalis*), *Rosmarinus*, *Lepechinia*, *Oreganum* and *Thymus*) [16]. The conversion of CA to carnosol under various stressful conditions has been noted [17], and exposure to high temperatures such as under cooking conditions can also lead to the production of a range of other structurally related phenols and quinones [18]. The two major diterpenes of rosemary (CA and CAR) have diverse pharmacological effects, but only their molecular pharmacology related to anti-inflammatory mechanisms of action is discussed in this communication. 

## 3. Methodology

Rosemary as well as its most abundant diterpenes CA and CAR are among the most investigated natural products. A literature search in PUBMED using the keyword “carnosic acid” returned 986 hits, and for “carnosol”, 393. The hit rate on ScienceDirect is higher at 2212 and 1706, respectively. For this review, the search hits were filtered by using additional keywords “inflammation”, “anti-inflammatory” and “anti-inflammatory” and included entries up to 10 January 2023. The findings were grouped under sections arranged by inflammatory mechanisms such as leucocyte activation, as leucocytes are among the major targets for anti-inflammatory drugs, and disease types such as lung, skin, cardiac, renal, hepatic, neuronal, endothelial, diabetes- and obesity-associated diseases and gut inflammation. The key findings associated with the outlined mechanisms of action are summarised in two tables as in vitro (Table 1) and in vivo (Table 2) outcomes. 

## 4. Mechanisms Related to Macrophages’ and Other Immune Cells’ Activation 

Macrophages play a key role in the immune response and can be induced to polarise into a pro-inflammatory phenotype by stimulation with a variety of agents such as bacterial lipopolysaccharide (LPS) and IFN-γ. This is a feature where they release pro-inflammatory cytokines such as IL-1, IL-6 and TNF-α, which are markers of chronic inflammatory diseases. They also release anti-inflammatory cytokines such as IL-10, which are relevant to the restoration stage of wound healing or the late stage of inflammation. The signalling cascades of these pro- and anti-inflammatory mechanisms and modulation by therapeutic agents are routinely assessed using macrophages and other immune cell cultures in vitro. These assessments are presented in this section along with some in vivo studies confirming the therapeutic potential of CA and CAR. 

Several studies employed the transformed cell line RAW 264.7 cells as a model of macrophage activation where the potential anti-inflammatory effect of rosemary diterpenes was evaluated. In the study by Oh et al. [19], CA (5–20 μg/mL) was shown to inhibit the release of nitric oxide (NO), TNF-α and prostaglandin E₂ (PGE₂) from stimulated RAW 264.7 cells. As a stimulus, they used Toll-like receptor (TLR)-2 ligands, Gram-positive-bacteria-derived peptidoglycan (PGN), pam3CSK (TLR2/TLR1 ligand) and the TLR4 ligand and Gram-negative-bacteria-derived lipopolysaccharide (LPS). CA blocked the nuclear translocation of NF-κB and its upstream signalling including Syk/Src, phosphoinositide 3-kinase (PI3K), Akt (protein kinase B), inhibitor of κBα (IκBα) kinase (IKK) and IκBα for NF-κB activation. Kinase assays revealed that Syk could be a direct enzymatic target for CA in its anti-inflammatory action. Meanwhile, a direct effect of CA on bacteria should not be ruled out as the compound arrested the growth of dermatitis-inducing Gram-positive and Gram-negative microorganisms such *Propionibacterium acnes*, *Pseudomonas aeruginosa* and *Staphylococcus aureus*. In the same concentration range (up to 10 μM), both CA and CAR were also shown to inhibit the secretion of matrix metallopeptidase 9 (MMP-9), and monocyte chemoattractant protein-1 (MCP-1) from LPS stimulated RAW 264.7 cells’ as well as TNF-α-stimulated rat vascular smooth muscle cells’ (VSMCs’) activity in a cell migration assay in vitro [20]. The production of NO from LPS-stimulated RAW 264.7 cells in vitro was also shown to be suppressed both by CA and CAR within the concentration range of 12.5–50 μg/mL [21]. Wang et al. [22] also investigated the signalling pathway associated with the anti-inflammatory activity of CA (2.5–20 μM) in LPS-stimulated RAW 264.7 cells. In addition to a reduction in the levels of NO and TNF-α, the compound also downregulated cyclooxygenase-2 (COX-2) protein expression, as well as the transcriptional level of inflammatory genes including *Nos2*, *Tnfα*, *Cox2* and *Mcp1*. Moreover, the mitogen-activated protein kinases (MAPKs) including extracellular signal-regulated kinase 1/2 (ERK), Jun N-terminal kinase (JNK) and p38, along with NF-κB, and FoxO signalling pathways, were suppressed, as evidenced by the inactivation of IKKβ/IκB-α/NF-κB, MAPKs and FoxO1/3. A similar study for CAR was conducted by Lo et al. [23] using the LPS-stimulated RAW 264.7 macrophage. Inhibition of NO production (IC_50_ of 9.4 μM) coupled with iNOS mRNA and protein expression, a reduction in NF-κB subunits’ translocation, and DNA binding activity of NF-κB were also observed. The downregulation of IKK activity by CAR (5 μM) was further shown to be associated with inhibition of LPS-induced phosphorylation as well as degradation of IκBα. As with the above-mentioned effect of CA, the LPS-induced p38 and p44/42 MAPK activation was also inhibited by CAR (20 μM). Other similar studies by Lee et al. [24] demonstrated the inhibitory effect of CAR (1, 2 and 5 μM) on LPS-induced NO and expression of iNOS and COX-2 in RAW 264.7 cells. In this case, inhibition of the phosphorylation of signal transducer and activator of transcription 3 (STAT3) and DNA binding activity in RAW 264.7 cells coupled with a docking model showing potential direct binding activity to the DNA binding domain of STAT3 was revealed. In addition, both CA and CAR within the concentration range of 5–15 μM reduced NO and prostaglandin E₂ (PGE₂) production in LPS-stimulated RAW 264.7. This activity was linked to inhibition of gene expression of iNOS, cytokines/interleukins (IL-1α, IL-6) and chemokines including CCL5/RANTES and CXCL10/IP-10, coupled with suppression of nuclear translocation of NF-κBp65, as evidenced in IL-1β-stimulated cells [25]. 

To study the anti-inflammatory properties of CAR in vitro, Shi et al. [26] employed primary mouse bone-marrow-derived macrophages (BMDMs), THP-1 cells and human peripheral blood mononuclear cells (hPBMCs). Their major finding was the inhibition (2.5–10 µM in a concentration-dependent manner) of NOD-like receptor family pyrin domain containing 3 (NLRP3) inflammasome activation by directly targeting heat-shock protein 90 (HSP90). This was coupled with inhibition of pro-inflammatory cytokine expression (pro-IL-1β, TNF-α and IL-6) by pre-treatment of hPBMCs with CAR prior to LPS induction. They also performed experiments in vivo where administration of CAR (20 or 40 mg/kg, i.p.) in mice could ameliorate the endotoxemia or IL-1β and TNF-α production induced by LPS, a process inhibited by an NLRP3 inflammasome inhibitor, MCC950. The ethanolic rosemary extract (as well as CA and CAR) were also shown to suppress the secretion and mRNA expression of IL-8, IL-1β and TNF-α in *Propionibacterium acnes*-stimulated monocytic THP-1 cells [76].

Studies both in human whole-blood stimulated with LPS, and a cell-free system also showed that CA and CAR did not affect the activity of COX-1 and COX-2 directly but modulated the activity of microsomal PGE_2_ synthase (mPGES)-1, an enzyme upstream of COX [27,28]. The differential effect of the two compounds in the cell system, however, need further clarification. As a model of allergic inflammatory reaction, Crozier et al. [29] employed a mast cell culture in vitro using anti-TNP IgE as a sensitising agent. They showed that CA (1.59–100 µM) could ameliorate the allergen-induced ROS generation, Ca^2+^ mobilisation and degranulation. Hand in hand with these effects were suppression of the release of pro-inflammatory cytokines (IL-6, IL-13 and TNF) and chemokines (CCL2, CCL3 and CCL9), based on their protein and gene expression levels, though the effects were demonstrated at moderate concentrations (50 and 100 μM). As a mechanism of action, CA (15 and 50 μM) reduced the phosphorylation of both IKK and IκBα while it also decreased NF-κB2 mRNA, a relevant gene for transcribing the p52/100 subunit of NF-κB. Other allergen-specific genes suppressed by the compound were *c-jun*, *Egr1* and *Egr2.* CA (15 and 50 μM) further inhibited the phosphorylation levels of Syk (Tyr352 and 525/526) and Akt, which are known to be involved in NF-κB-pro-inflammatory signalling. Of the MAPKs affected was the upstream TAK1 (Ser412), which is known to be relevant to allergic reactions, but unlike activities reported from macrophages, the phosphorylation of MAPKs ERK, JNK and p38 in primary cultures of mast cells was not affected. Hence, the major signalling affected by CA in allergic inflammation induced in mast cells appears to be inhibition of Syk activity and phosphorylation, TAK1 (Ser412), Akt (Ser473) and upstream NF-κB signalling [29]. The studies outlined in this section overall revealed the dual antioxidant and anti-inflammatory properties of CA and CAR. These effects are in line with our knowledge of rosemary extract’s role in ameliorating allergen-mediated mast cell activation [77]. 

The macrophage equivalent cell system in the CNS, operated by microglia, also appears to be modulated by rosemary diterpenes. The study by Foresti et al. [30] using BV2 mouse microglial cells treated with CA or CAR (5 μM) revealed induction of HO-1 coupled with inhibition of the LPS and INF-γ-induced NO and TNF-α, and PGE_2_ production. In BV2 cells subjected to oxygen-glucose deprivation (OGD), CAR decreased the levels of malondialdehyde (MDA), lipid peroxidation (LPO), TNF-α, IL-1β and IL-6, and increased the levels of the reduced form of glutathione (GSH), superoxide dismutase (SOD), IL-4 and IL-10 [30,78]. Readers should bear in mind that this effect is mainly an activation of a survival mechanism by CAR under stress conditions via the PI3K/Akt/mTOR signalling pathway as selective inhibitors antagonise the positive effect of CAR in these cells. Furthermore, these data are in line with our understanding of this pathway as a survival mechanism in cancer cells and a means of their resistance to chemotherapy [79]. 

## 5. Arthritis

Hosokawa et al. [31] employed IL-1β- or TNF-α-stimulated human periodontal ligament cells to study the anti-inflammatory effect of CA. They showed that the compounds could effectively suppress the release of IL-6 and production of CXC chemokine ligand (CXCL)10, CC chemokine ligand (CCL)2 and CCL20. As demonstrated in the macrophage system, the compounds could also suppress the JNK, NF-κB and STAT3 pathways of activation induced by IL-1β or TNF-α. These data are consistent with the authors’ previous report using human oral epithelial cell line (TR146) cells stimulated by IL-27, where CA (3.125–50 µM) showed a suppressive effect on chemokine (CXCL9, CXCL10 and CXCL11) production along with significant inhibition of the phosphorylation of STAT1, STAT3 and Akt [32]. In an adjuvant arthritis model in rats, administration of methotrexate (0.3 mg/kg) in combination with CA (100 mg/kg, p.o.) for up to 28 days was shown to suppress hind paw swelling, the levels of IL-17A, MMP-9 and MCP-1 in plasma, and gamma-glutamyltransferase (GGT) activity in joint homogenates [52]. As a further mechanism of action, the mRNA expression levels of HO-1 and catalase (CAT) were increased, while IL-1β was reduced in the liver by the drug combination but not individual components alone. 

Liu et al. [33] used an in vitro (osteoclasts and fibroblast-like synoviocytes) and collagen-induced arthritis experimental model in rats to study the anti-inflammatory potential of CA. The pro-inflammatory proteins suppressed by the compound included TNF-ɑ, IL-1β, IL-6, IL-8, IL-17, MMP-3 and receptor activator for nuclear factor-κB ligand (RANKL). Along with the observed inhibition of osteoclastogenesis and joint destruction, the RANKL-induced activation of NF-κB and MAPKs (JNK and p38) leading to the downregulation of NFATc1 (plays a role in the inducible expression of cytokine genes) was also ameliorated by the compound. In a collagen-induced arthritis db/db mice model of rheumatoid arthritis, Xia et al. [53] demonstrated improvement in bone loss by CA (30 and 60 mg/kg, i.p. daily for 4 weeks) along with modulation of the fasting blood glucose and glucose levels in an oral glucose tolerance test (OGTT) and insulin tolerance test (ITT). In vitro (bone marrow cells and osteoblasts), CA (10 or 20 μM) suppressed (RANKL)- and macrophage colony-stimulating factor (M-CSF)-induced osteoclastogenesis. Among the oxidative stress and inflammation markers suppressed both in vitro and in vivo were ROS (while upregulating SOD and glutathione peroxidase (GPx) activity) the RANKL- and M-CSF-induced p38 MAPK, NF-κB phosphorylation and cytokine (TNF-α, IL-1β and IL-18) and COX2 expression [53]. 

Li et al. [54] used a type II collagen-induced arthritis DBA/1 model in mice to study the potential effects of rosmanol (40 mg/kg/d, p.o.) and CAR (40 mg/kg/d, p.o.) alone. The compounds could alleviate rheumatoid arthritis symptoms (swelling, redness and synovitis; decreased the arthritis index score) along with the serum level of pro-inflammatory cytokine (IL-6, MCP-1 and TNF-α). Other inflammation markers blocked by the compounds were the TLR4/ NF-κB/JNK and p38 MAPK pathways in synovial tissue. In a drug combination study, a 20 mg/kg dose of each resulted in higher activity than individual compounds, suggesting a possible additive/synergistic anti-inflammatory effect. 

In the chondrosarcoma cell line SW1353 and in primary human chondrocytes, CA and CAR, at a concentration range of 5–15 µM, inhibited IL-1β-induced catabolic genes such MMP-13 and ADAMTS-4. These downregulated genes contributed to cartilage erosion, while the expression of anabolic genes including Col2A1 and aggrecan was shifted by the compounds toward the pre-pathophysiological homeostasis state. The induced nuclear translocation of NF-κBp65 was also inhibited [25]. Overall, CA and CAR appear to suppress the arthritis inflammatory score and markers including NF-κB, MAPK, STATs, pro-inflammatory cytokines, chemokines and matrix degradation by lowering the MMP level. 

## 6. Lung Inflammation

Direct evidence for the therapeutic potential of CA in alleviating lung inflammation came from the study by Tsai et al. [34] both in vitro and in vivo. Human neutrophils primed for respiratory burst (superoxide anion and ROS release) with *N*-formyl-L-methionyl-L-leucyl-L-phenylalanine (fMLF) (FPR1 agonist), MMK1 (FPR2 agonist) and PMA (protein kinase C activator) showed a reduced level of respiratory burst when treated by CA (1–10 μM). As a measure of inflammation, the fMLF-stimulated expression of integrin adhesion molecules (CD11b) and neutrophil adhesion to the surface of endothelial cells (bEND 3 cells) were suppressed at the same concentration range through a mechanism associated with inhibition of phosphorylation of MAPKs (ERK, JNK and p38). The in vivo experimental model employed in the study was acute respiratory distress syndrome (ARDS) in mice induced by LPS spray into the trachea [34]. In this case, administration of CA (5 or 10 mg/kg, i.v.) alleviated the symptoms as assessed by qualitative (histology) and quantitative (MPO activities, immunohistochemistry and immunofluorescence staining) assays. The oxidative state and neutrophil infiltration level, as assessed using anti-Ly6G (targeting the component of the myeloid differentiation antigen) and anti-4-HNE (targeting the lipid peroxidation product, 4-hydroxy-2-nonenal) antibodies, suggested the anti-inflammatory properties of CA [34]. An LPS-induced acute lung injury (ALI) experimental model in mice was also used to study the effect of CA (10, 20 and 40 mg/kg doses, i.p.). A protective effect was evidenced by histologic results and a reduction in the wet-to-dry ratio of lung tissues, while an anti-inflammatory effect was evident from the suppression of neutrophil apoptosis, and production (mRNA and protein) of IL-1β, IL-6, TNF-α, TLR4 and NF-κB expression, as well as of NF-κB phosphorylation in lung tissues [55]. When bleomycin was used to induce lung damage in rats, CAR (at doses of 10, 20 and 40 mg/kg, p.o.) was shown to reduce the levels of oxidative markers (MDA, NO, protein carbonyl) and pro-inflammatory cytokines (TNF-α and IL-6 levels) and the myeloperoxidase (MPO) activity in the lungs. Meanwhile, antioxidant markers (GSH content, catalase, GPx and SOD activities) were increased in line with the improvement of lung fibrosis and histopathological changes [56]. Lee and Im [57] used an ovalbumin-induced allergic asthma experimental model in mice to reveal the effect of CAR (5 mg/kg, i.p.). In addition to suppressing the increase in the number of eosinophils in the bronchoalveolar lavage fluids, cytokine production, including IL-4 and IL-13, was also suppressed in both bronchoalveolar lavage fluids (BALF) and the lungs. These findings are consistent with the known suppressive effect of rosemary extract on allergic airway inflammation [80]. 

In human lung NCI-H1975 cells, CAR (3 μM) upregulated the HO-1 level and protected cells from H_2_O_2_-induced cell death. It also protected lung tissues in an excised-lung organ culture ischemia model [35]. This ex vivo culture was sourced from mice treated with carnosol-enriched *Callicarpa longissima* extract (30 mg/kg, p.o.), which was associated with upregulation of HO-1 expression. 

## 7. Skin Inflammation 

Mengoni et al. [21] used a phorbol 12-myristate 13-acetate (PMA)-induced ear inflammation model in mice where CA and CAR reduced oedema with EC_50_ values, respectively, of 10.20 μg/cm^2^ and 10.70 μg/cm^2^. The inhibition of leucocyte infiltration and epidermal ulceration induced by PMA were also coupled with reduction in the skin tissue expression levels of IL-1β, TNF-α and COX-2 (not COX-1), and to a less extent, fibronectin, and ICAM-1 expression. The carrageenan-induced mouse hyperalgesia model in the hind paw is one of the most commonly used anti-inflammatory assays in vivo. In one study, in a mouse model of pleurisy induced by carrageenan, both the crude rosemary plant extract as well as CAR and rosmarinic acid decreased the pro-inflammatory markers (MPO, adenosine-deaminase, NO and IL-17A) while increasing the anti-inflammatory cytokine, IL-10, level [58]. In an in vivo mouse model of *P. acnes*-induced ear swelling and granulomatous inflammation, the crude extract of rosemary was shown to alleviate inflammation [76].

In a mouse atopic dermatitis experimental model induced by 5% phthalic anhydride, CAR (0.05 µg/cm^2^) was shown to suppress skin inflammation along with inhibition of the expression of iNOS and COX-2 in skin tissue [24]. This activity was coupled with inhibition of the activation of STAT3 in skin tissue, while in the blood serum, the levels of TNF-α, IL-1β and immunoglobulin-E were also suppressed. The experimental model employed the administration of CAR (0.05 μg/cm^2^) together with an inflammatory inducer, followed 3 h later by 100 μL (20 μL/cm^2^) of 10 μM CAR. Skin inflammation can also be induced by exposure to UVB, in which case CAR also demonstrated efficacy, as reported by Yeo et al. [59]. Topical application of CAR (0.05 µg/cm^2^) on UVB (540 mJ/cm^2^, for 3 successive days)-induced skin inflammation in HR1 mice was shown to reduce erythema, epidermal thickness and inflammatory responses such as reduction in the levels of immunoglobulin-E and IL-1β in blood serum. The suppressed inflammatory markers included iNOS and COX-2 in the back skin, coupled with decreased activation of STAT3 and its upstream signal, Janus kinase (JAK). By using the carrageenan-induced oedema model, Maione et al. [28] demonstrated that CAR and CA (30 or 100 µg per paw) displayed a dose-dependent anti-inflammatory effect (oedema) and suppressed microsomal prostaglandin E synthase-1 (mPGES-1)- and 5-LO-derived products. 

Oh et al. [19] studied an in vitro inflammatory skin model and showed that CA (5– 20 μg/mL) could suppress the production of IL-6, IL-8 and MCP-1 in keratinocyte HaCaT cells stimulated with sodium lauryl sulphate (SLS) and retinoic acid (RA). 

## 8. Neuroinflammation

The direct anti-inflammatory effect of test compounds can be assessed in neuronal cultures in vitro. By using the SH-SY5Y cells exposed to paraquat, de Oliveira et al. [36] showed the neuroprotective effect of CA (1 μM) through anti-inflammatory mechanisms such as reduction in the production of IL-1β, TNF-α and COX-2. They also showed that the Nrf2 and HO-1 signalling pathway of cytoprotection was activated while the activation of the NF-κB transcription factor was suppressed by CA. In the neuronal cell line of PC12 cells subjected to serum starvation, CAR (10 µM) was shown to increase the induction of HO-1 (protein level) and increased Nrf2 expression [37]. This survival mechanism induced by CAR, unlike that described for LPS (Section 4) and other pro-inflammatory agents’ stimulation, involves activation or phosphorylation of the MAPKs (ERK, p38, JNK, Akt and its downstream effector PI3K). Large volume of literature is available in demonstrating the neuroprotective effect of rosemary diterpenes through antioxidant mechanisms. For example, CA as a potential therapeutic agent for treating Parkinson’s disease was shown by [38] to ameliorate 6-hydroxydopamine (6-OHDA)-induced neuronal death both in vitro (in SH-SY5Y cells, 1 µM) and in vivo in rats (20 mg/kg, p.o.). Beyond reversing the behavioural changes, LPO was reduced while GSH level and SOD activity were enhanced. Moreover, cell apoptosis induced by 6-OHDA via activation of the MAPKs was inhibited, as evidenced by suppression of the phosphorylation of JNK and p38. This was also effectively demonstrated in PC12 cells subjected to hypoxia-induced neuronal damage where CA ameliorated inflammation, oxidative stress and cell death [39].

When neuroinflammation was induced in mice by exposure to organophosphate pesticide (chlorpyrifos), daily administration of CA (30 and 60 mg/kg p.o. for 14 days) was shown to ameliorate the biochemical changes in a dose-dependent manner. This included reversal of the increased serum concentrations of pro-inflammatory cytokines (IL-1β, IL-6 and TNF-α), a reduced level of acetylcholinesterase (AChE) and antioxidant markers (GSH, GPx, SOD and CAT) and an increased level of prooxidant (MDA and NO) markers in cerebral and ocular tissues [60]. 

Maynard et al. [61] employed repetitive mild traumatic brain injury (TBI) in mice, where CA at a small dose (1 mg/kg, i.p.) was shown to alleviate neuronal damage. By activating the Nrf2 pathway of the antioxidant/anti-inflammatory mechanism, the compound further suppressed the transcription factor, NF-κB. Another similar neuroprotective study was that by Wang et al. [62], which demonstrated that CAR protects against spinal cord injury through Nrf2 upregulation. The oxidative stress markers of increased ROS generation, total oxidant levels, LPO content, protein carbonyl and sulfhydryl levels were also suppressed, along with the expression of NF-κB and COX-2, while the disease-associated or -altered phosphorylated Akt and Nrf2 levels were reversed by the compound. The neuroprotective effect of CA (0.3, 1.0 or 3.0 mg/kg, i.p.) in traumatic brain injury in mice was also shown to be mediated through activation of the Nrf2–ARE pathways [63]. Hence, oxidative markers were reduced while mitochondrial respiratory dysfunction and neuronal damage, as shown by cytoskeletal damage and biochemical markers’ (4-HNE (lipid peroxidation) levels in the hippocampus and cortex and 3-NT (protein nitration) in the cortex, were reduced by the compound. 

Teng et al. [64] used the subarachnoid haemorrhage (SAH) of early brain injury model, where CA displayed a protective effect. Among the improved parameters were decreasing ROS levels, brain oedema and blood–brain barrier permeability, neuronal cell death and neuronal function improvement. Furthermore, CA was shown to increase the SIRT1, MnSOD and Bcl-2 expression while apoptosis markers were suppressed. More studies in this line showing the anti-inflammatory effect of the compound are needed. In the APP/PS1 mouse model of Alzheimer’s disease, administration of CA was shown to reduce β-amyloid (Aβ) deposition and ameliorate cognitive impairment and pro-inflammatory cytokine (IL-1β, TNFα and IL-6) production [65]. By blocking the interaction of CEBPβ with NF-κB p65, the transcription of the NF-κB target genes TNF-α and IL-6, as well as Aβ secretion, were suppressed. A good example of neuroinflammation is that of experimental autoimmune encephalomyelitis (EAE), an animal model of multiple sclerosis (MS). Li et al. [66] showed that CAR could reduce demyelination, inhibit Th17 cell differentiation and STAT3 phosphorylation and block the translocation of NF-κB. The compound could also switch the inflammatory phenotypes of infiltrated macrophage/microglia in the chronic stage of the disease. 

In BV2 microglial cells in vitro, CAR (5–20 μM) was one of the compounds identified to induce HO-1 and Nrf2, thereby inhibiting the production of TNF-α, PGE_2_ and NO stimulated by interferon-γ (INF-γ) or (LPS) [30]. The anti-inflammatory effect in the neuronal system could also mark an indirect effect via inhibition of amyloid beta (Aβ) production, as shown for CA (30 µM). It ameliorated Aβ (1-40 and 1-42) production by activating α-secretase TACE (tumour necrosis factor-α-converting enzyme or disintegrin and metalloproteinase-17 or ADAM17) in cultured SH-SY5Y human neuroblastoma cells [40]. The selectivity of this enzyme was demonstrated as no effect was observed in β-secretase BACE1. It (50 μM) also suppressed the secretion and release of Aβ peptides (1-40, 1-42 and 1-43) in U373MG human astrocytoma cells by increasing the mRNA expression of an α-secretase (*TACE*) without affecting other secretases (*BACE1* and *PS1*) [40]. At the same time, increases in the HO-1 mRNA level were also observed in this experiment, suggesting the neuroprotective potential of the compound from inflammation and oxidative stress. 

Other neuronal effects of these compounds include the antinociceptive effect of CAR (10 mg/kg, p.o.) to chemical agents (acetic acid, formalin, glutamate, capsaicin and cinnamaldehyde) [81], formalin-induced pain response of CAR [82] and anti-inflammatory and anti-nociceptive effects of CA and CAR [28]. The overall trend thus seems to indicate that neuroprotection and inhibition of neuroinflammation are enacted by CA and CAR in a variety of experimental models. 

## 9. Diabetes-Associated Inflammation

Several studies investigated the antidiabetic potential of rosemary extracts, and indeed, the plant was shown to reduce the glucose level both in healthy and diabetic experimental animals [83,84,85,86,87]. Similarly, rosemary extract and diterpenes including CA and CAR were shown to increase insulin sensitivity, alleviate insulin resistance and protect cells from high-glucose-induced damage both in vitro and in vivo [88,89,90,91]. Hand in hand with this potential antidiabetic effect, the anti-obesity and lipid-lowering potential of rosemary and its phenolic diterpenes were established [71,92,93,94,95,96]. Given both diabetes and obesity are associated with inflammation, the anti-inflammatory mechanism of these compounds in such pathological conditions is worthy of scrutiny. Administration of CAR (1, 5, 10 mg/kg, i.p. for 4 weeks) in STZ-induced diabetic animals was shown to suppress the serum levels of glucose, IL-6, TNF-α, MDA, TG, TC, LDL-C, GST, SOD, CAT and HDL-C in a dose-dependent manner [67]. Hence, the compound could reduce the diabetes-associated increases in blood glucose level, oxidative stress and inflammation. The study by Ou et al. [68] also showed that CA (30 mg/kg) not only reduced the glucose level in STZ-induced diabetic rats but also the inflammation score. The study by Xie et al. [69] using the same diabetic model also corroborated the antidiabetic potential of CA (30 mg/kg, p.o.), which also involves suppression of the NF-κB activation. In 3T3-L1 adipocytes, CA (1–20 µM) reversed the TNF-α-mediated insulin resistance, as shown by insulin-stimulated glucose uptake and the phosphorylation of Tyr(632) insulin receptor substrate-1 (IRS-1), Akt and FoxO1 levels [42]. As an anti-inflammatory compound, it also attenuated the TNF-α-induced mRNA expression of inflammatory genes, including IL-6 and MCP-1. Furthermore, CA attenuated the TNF-α-mediated activation of ERK and JNK, the phosphorylation of inhibitor-κB (IκB) kinase (IKK)α/β, the phosphorylation and degradation of IκBα, the nuclear translocation of p65 and the DNA-binding activity of NF-κB and AP-1 [42].

## 10. Cardiac Inflammation

Hu et al. [70] employed the ischaemia/reperfusion model in diabetic mice on a high-fat diet under 30 min occlusion of the anterior descending coronary artery followed by reperfusion of the heart for 3 or 24 h. They showed that pre-treatment with CA (50 mg/kg, p.o.) could suppress the overproduction of ROS and pro-inflammatory cytokines (IL-6 and TNF-α). 

The cytoprotective effect of CA was demonstrated by the doxorubicin (DOX)-induced cardiotoxicity in rats at the dose of 10 mg/kg (p.o.) as well as in isolated rat cardiomyocyte (2.4–10 µM) cell cultures [43]. In addition to suppression of the ROS and NO levels, the DOX-induced expression of phospho-p38 and phospho-JNK1 proteins as well as NF–κB (p65) were lowered while the DOX-induced downregulation of Nrf2 in the nucleus and HO-1 in the cardiomyocytes were reversed. Similarly, the study by Zhang et al. [44] investigated using a cardiac muscle cell line, H9C2 cells, in vitro (5–20 μM), and the DOX-induced cardiotoxicity mice in vivo (5 mg/kg, p.o.) treated with CA or carvedilol. They showed a protective effect partly by augmenting the expression and activities of the antioxidant enzymes. In addition, the inflammatory response was significantly suppressed by the two compounds in combination, as shown by suppression of the levels of pro-inflammatory cytokines (TNF-α, IL-6, IL-1β and IL-18) and COX-2, which was associated with the inactivation of NF-κB. The effect of CAR (5–20 μM) on the LPS-stimulated cardiomyocyte cell line (H9C2) was also studied [45]. The major finding was the inhibition of the NF-*κ*B, and the NF-*κ*B-dependent inflammatory pathway associated with cytokine (TNF-*α*, IL-1*β*, IL-6) and COX-2 (as well as PGE_2_) expression. Their in silico analyses further suggested potential interaction of the compound with the binding site of the catalytic domain of IKK*β*. 

## 11. Hepato- and Renal Inflammation

The experimental models employed to demonstrate the hepatoprotective effect of CA (10 µM) include a chronic alcoholic liver injury model in rats (15 or 30 mg/kg, i.g.) and an in vitro model using HepG2 cells [46]. One major observation to note was the activation of SIRT1 to account for the antioxidant and anti-inflammatory response, as well as pathological markers of liver cell damage. The level of MnSOD was increased, while NF-κB and the serum level of TNF-α were inhibited. In an ischemia/reperfusion model of liver damage, Li et al. [47] demonstrated that nanoparticle preparations of CA (10 and 20 mg/kg, i.p.) could protect from liver injury progression when coupled with antioxidant (normalising the levels of SOD, CAT, GSH and GPx) and NF-κB signalling pathways of pro-inflammatory cytokine (TNF-α and IL-1β) expression. The in vitro experiment in the same study, using LPS-treated hepatic stellate cells, also showed that CA nanoparticles could inactivate phosphorylated IKKα, IκBα and NF-κB, leading to decreased TNF-α, IL-1β and IL-18 expression. 

In a high-fat-diet (HFD)-induced non-alcoholic fatty liver disease (NAFLD) model in mice, the pharmacological activity of CA (15 mg/kg, p.o.) was reported to be related to an improvement in lipid metabolism and inflammation markers [71]. Notably, the serum and hepatic tissue levels of IL-1β, IL-18, TNF-α, IL-2, IL-4, IL-6, IL-12 and IFN-γ were suppressed. The link between these activities and myristoylated alanine-rich C-kinase substrate (MARCKS) via the PI3K/Akt, NLRP3/NF-κB and SREBP-1c signalling pathways of inflammation was established using inhibitors and MARCKS-deficient mice. Another interesting finding both for the antioxidant and anti-inflammatory effect of CA came from a study by Xiang et al. [72] of LPS-induced liver injury in rats. Administration of CA (30 or 60 mg/kg, p.o.) alleviated symptoms of liver damage, as assessed by histology and biochemical markers (alanine aminotransferase, aspartate aminotransferase and alkaline phosphatase). In addition to suppression of immigration of inflammatory cells, the serum levels of TNF-α and IL-6 were suppressed. The oxidative markers suppressed were NO and ROS, while antioxidant levels (SOD, GSH and GPx) in the serum and liver were augmented. 

Administration of CA (40 mg/kg, i.p.) in mice after LPS injection was shown to ameliorate histological abnormalities and renal dysfunction [73]. Notably, the pro-inflammatory cytokine (IL-1β, IL-6, TNF-α and MCP-1, mRNA and protein level) expression, immune cell (neutrophil) infiltration and NF-κB activation induced by LPS injection were suppressed by CA. Among the oxidative markers improved by the compound were GSH, NOX4 (mRNA and protein), CAT and MnSOD, which were associated with reduced tubular cell apoptosis. Other data also suggest the renal protective effect of CA, such as those demonstrated by Xie et al. [69]. In STZ-induced diabetic db/db mice, a nephroprotective effect of CA (15 or 30 mg/kg, i.g.) associated with activation of Nrf2 and inhibition of NF-κB was demonstrated. On the other hand, the experiment by Zheng et al. [74] suggested that CAR (3 mg/kg) protects against renal ischemia-reperfusion injury in rats when administered intravenously. The inhibition of apoptotic tubular cell death, caspase-3 activation and activation of the p38 pathway was evident. To study the anti-inflammatory properties of CAR (20 or 40 mg/kg, i.p.), Shi et al. [26] employed an in vivo non-alcoholic steatohepatitis model in mice, which was shown to be associated with downregulation of IL-1β and TNF-α. This activity was associated with suppression of NLRP3 inflammasome activity via a direct effect on heat-shock protein 90 (HSP90), as explained in Section 4 for a macrophage culture in vitro. 

## 12. Obesity-Associated Inflammation 

In a-high fat-induced mouse obesity and metabolic syndrome model, CA treatment (10 or 20 mg/kg, p.o.) was shown to decrease the serum levels of triglycerides, total cholesterol, insulin and glucose [75]. As an anti-inflammatory compound, CA decreased the protein expression levels of various pro-inflammatory cytokines (IL-1β, IL-6 and TNF-α) in serum and brain tissues, along with suppression of the NF-κB signalling pathway. As a cytoprotective agent, it promoted the expression levels of anti-apoptotic Bcl-2, while decreasing the levels of pro-apoptotic Bax and matrix metallopeptidase 9 [75].

In 3T3-L1 adipocytes stimulated by LPS, the TLR4-mediated elevated mRNA expression of TNF-α, IL-6 and MCP-1 was suppressed by CA (up to 20 µM). In addition to the LPS-induced upregulation of TLR4, myeloid differentiation factor 88, TNF receptor-associated factor 6 and ERK were also suppressed by CA [48]. These data were in line with the study by Tsai et al. [42], which showed that 3T3-L1 adipocytes stimulated with TNF-α could be targeted by CA, leading to reduced levels of mRNA expression of inflammatory genes (*IL-6* and *MCP-1*). Among the signal transduction pathways affected by CA in this study were the activation of ERK and JNK; the phosphorylation of IκB, (IKK)α/β; the phosphorylation and degradation of IκBα, the NF-κB p65 subunit; and the DNA-binding activity of NF-κB and AP-1. The modulation of diabetes, obesity and metabolic syndrome by rosemary extract and diterpenes is also a result of the effect on various other metabolic signalling pathways such as PPAR-γ and AMPK [91,94,96,97].

## 13. Endothelial Inflammation

The study by D’Agata et al. [49] employed an in vitro culture of human retinal endothelial cells to study high-glucose-induced ROS generation and cell damage and death. By upregulating the expression and activity of Nrf2, HO-1 and ERK1/2, CAR was demonstrated to have a cytoprotective effect in endothelial cells. In addition to increasing the expression of endothelial cadherin (VE-cadherin), thereby improving the integrity of intercellular junctions, CAR (10 µM) was also shown to protect human lung microvascular endothelial cells (HMVEC-L) from tert-butyl hydroperoxide (t-BHP)-induced cell death [50]. The antioxidant mechanism of action was evident from the increased expression of Nrf2 and HO-1, and it also interrupted the Nrf2-Keap1 protein−protein interaction. 

## 14. Gut Inflammation

Xu et al. [51] employed a dextran sulphate sodium (DSS) experimental model of colitis in mice. They showed that induction of an improvement in the clinical symptoms and colonic pathological damage by CAR (50 mg/kg i.p. for 10 days) was associated with reduction in inflammatory cell infiltration and cytokine (TNF-α, IL-1β, IL-6 and IFN-γ) expression. This evidence was further substantiated in vitro (10 µM) using thapsigargin-induced endoplasmic reticulum (ER) stress in HCT-116 cells (an intestinal epithelial cell line) as well as colonic mucosa tissues (2–3 pieces from patients). As with the in vivo data, suppression of pro-inflammatory mediators (TNF-α, IL-6, IFN-γ and CXCL10) was evident in the in vitro assay. Although the main emphasis was on anticancer effect analysis of CA, Li et al. [98] also demonstrated that CA suppressed the inflammatory response in colorectal cancer in mice by reducing the levels of IL-1β, -6 and -17A. Readers should also note that the protective effect of these diterpenes in the gut could be associated with modulation of the microbiota structure and population, as reported under various experimental conditions [68,95,98,99]. 

## 15. General Discussion

As a defence mechanism to protect cells and organs from damage induced both from internal and external sources, inflammation is universally involved in disease processes, either at the initiation phase or later stages of pathologies. Anti-inflammatory drugs of the classical steroidal class target phospholipase enzymes, which catalyse the initial step in the synthesis of lipid mediators such as prostaglandins, and leukotrienes. They also have various other mechanisms including suppression of cytokine expression and serve in therapeutics associated with immunosuppression (e.g., organ transplantation). We also have classical examples of small molecules of anti-inflammatory agents that target the active site of enzymes such as COX (selective new generation and non-selective aspirin type) and lipoxygenases. As highlighted in the introduction section, these drugs of high significance to acute inflammatory conditions are, however, of little benefit for chronic inflammatory diseases such as those highlighted in these articles. In this case, antibody approaches targeting pro-inflammatory cytokines, their receptors or effector proteins such as adhesion molecules have been effectively employed in recent years. Given the challenge of using protein-based drugs, the search for a small-molecular-weight antagonist of protein mediators for chronic inflammation therapy is ongoing. Unfortunately, small molecules have little impact on ligand–receptor interaction of the protein–protein type. Meanwhile, the signal transduction pathways of protein signalling molecules (e.g., cytokines) are subject to modulation by small-molecular-weight compounds including natural products. The crosstalk of signalling between inflammation and oxidative stress also creates opportunities to utilise natural products, which are commonly regarded as antioxidants. 

In various sections of this article, the antioxidant potential of rosemary diterpenes was shown through experimental evidence where the production of ROS, lipid peroxidation products (e.g., MDA) and NO was reduced while antioxidant defences such as the GSH level and activity of antioxidant enzymes (SOD, CAT, GPx) were augmented. While ROS-generating systems such as NOX4 are inhibited, antioxidant defences including NQO-1 and Nrf2/HO-1 are activated. Hand in hand with these antioxidant effects, the anti-inflammatory activity of CA and CAR was demonstrated through experimental evidence showing suppression of pro-inflammatory markers under several disease models. The common links in these diseases, constituting anti-inflammatory mechanisms at the molecular level of signalling, are summarised in the following sections.

### 15.1. Rosemary Diterpenes Inhibit Activation of NF-κB

**Figure 2:**
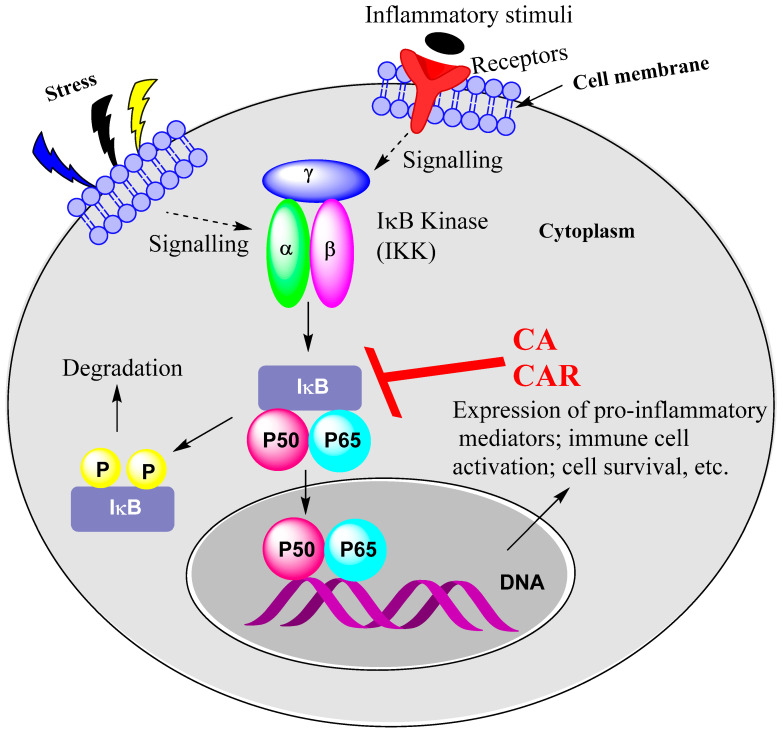
NF-κB activation as a target for CA and CAR. Stress and inflammatory mediators and growth factors, PAMPs and cytokines such as TNF, IL-1 and IL-6 all activate NF-κB. Their relevant receptors include growth factor receptors, TLRs and cytokine receptors such as TNF receptor. CA and CAR have been shown to suppress the activity of IKK, leading to inhibition of NF-κB activation and/or mobilisation to the nucleus. Upstream signalling pathways could also be a target, as shown for CA, inhibiting the TLR-mediated activation of Syk/Src-PI3K and thereby abolishing the LPS-induced IKK activation.

In various sections of this article, the anti-inflammatory properties of CA and CAR have been shown to be associated with inhibition of upregulation of NF-κB under inflammatory conditions. The expression of pro-inflammatory genes leading to cytokines, chemokines, adhesion molecules and enzymes (e.g., COX) for lipid mediators are all under the control of the transcription factor, NF-κB. Inevitably, suppression of inflammation-mediated upregulation of NF-κB inhibits immune cell activation and the inflammatory score under various disease conditions, as highlighted in this article. Readers who would like more insight into the role of NF-κB in various inflammatory diseases should refer to review articles in the field [100,101]. Various physical and chemical pressures, pro-inflammatory cytokines and bacterial and viral products are known to stimulate the activation of NF-κB. As shown for CA and CAR, the common target for inhibiting NF-κB is by modulating a family of inhibitory molecules (IκBs) such as IκBα. The NF-κB proteins (p65/RelA, RelB, c-Rel, p50 and p52) that form dimers are retained in the cytoplasm in their inactive form as they exist in association with the IκB proteins. The NF-κB activation cascade thus involves inactivation of the inhibitory proteins through phosphorylation and their subsequent degradation. The IκB kinase complex (IKK with IKK1/α and IKK2/β subunits and regulatory subunit IKKγ) that does this task is the common target for anti-inflammatory compounds, as shown in this article for CA and CAR (Figure 2). The multiple effects of these compounds in the NF-κB signalling pathways were demonstrated in the study by Oh et al. [19], which showed inhibition of the LPS-mediated and TLR-dependent NF-κB activation by CA was associated with Syk/Src, PI3K and Akt inhibition. The critical role of these pathways in NF-κB activation via IKK was demonstrated through inhibitor studies and various inflammatory disease models [102,103,104]. Many natural products also display anti-inflammatory activities by suppressing NF-κB activation in a similar manner. These include curcumin [105,106,107], gallate derivatives [108], quercetin [109] and resveratrol [110,111], among others. 

### 15.2. Rosemary Diterpenes Modulate the MAPK Pathways

**Figure 3:**
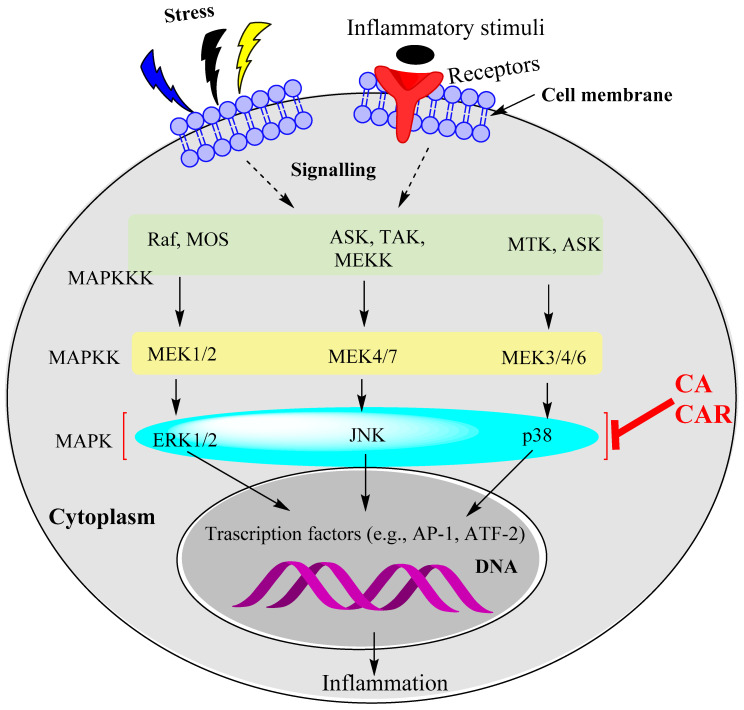
The MAPK pathways as targets for CA and CAR. The evidence available so far suggests that ERK1/2, JUNK and p38 MAPK phosphorylation are targeted by rosemary diterpenes to induce anti-inflammatory effects. Further research is required to determine whether the other kinases upstream of MAPKs are affected by CA and CAR. At least one study shows that upstream kinases (TAK1) could also be affected.

The role of the MAPK pathways in inflammation has been well-established but complexity arises from these pathways also being involved in various other cellular processes such as proliferation, differentiation and cell death (apoptosis). The processes are based on phosphorylation steps in a sequence involving MAPK kinase kinase (MAPKKK), MAPK kinase (MAPKK) and MAPK. The MAPKs, in turn, activate via phosphorylation several other enzymes as substrates called MAPK-activated protein kinases (MAPKAPKs). This level of complexity was not demonstrated in studies using CA and CAR, and hence only the relevant MAPKs are described herein. The three most common MAPKs relevant to inflammation are ERK 1/2 (mostly described as ERK, though we also have ERK 3/4, ERK5 and ERK7/8), JNK and p38 MAPK. Various isoforms of these MAPKs are also known, for example, α, β, γ and δ isoforms of p38. As shown in the various sections of this article, these three MAPKs are involved in inflammation induced by a variety of agents (cellular stress, growth factors, PAMPs), pro-inflammatory cytokines and cell survival cases such as neurons subjected to cellular stress from glucose deprivation or direct physical damage. A simplistic presentation of the MAPK pathways is shown in Figure 3, where they all culminate in the activation of transcription factors such as AP-1 and others. The pathways also have a crosstalk with NF-κB activation. For example, activation of MAPKKK (e.g., TAK1) leads to NF-κB activation via IKK phosphorylation. For details of the MAPK signalling pathways, readers are directed to review articles in the field [112,113,114]. The synthesis of pro-inflammatory cytokines involves activation of the MAPKs, and hence the suppressive effect of CA and CAR on this signalling system should be considered as one of their mechanisms of anti-inflammatory effect. Cell survival as neuronal and cardiac cells are damaged under stress also requires activation of these pathways, and hence CA and CAR paradoxically may activate the MAPKs as part of their cytoprotective effect. Meanwhile, the MAPKs are also key targets in cancer of various agents [115] including CA. The toxicity of therapeutic agents and cell death in cardiac cells (e.g., by doxorubicin) was shown to be associated with pro-inflammatory cytokine expression linked to upregulation of the MAPK pathway (e.g., [116]). The association of doxorubicin toxicity in cardiac cells with p38 MAPK overactivity was also established (e.g., [117]). While suppressing MAPKs’ activity has been researched as a key target for therapeutic intervention in cancer and inflammation, their role in antiapoptotic events, once again, is emerging as a complication in adopting such a therapeutic approach. In this line, the study by Martin et al. [37] showing activation of the MAPKs by CAR as a mechanism in neuroprotection is interesting. The pro- and anti-apoptotic roles of the MAPKs have also been reviewed [118]. In addition, it is worth noting that CA displayed antiproliferative and cancer metastasis inhibitory effects by inhibiting the ERK, p-38 and JNK signalling pathways [119]. Hence, further research is needed to establish the link between MAPK signalling and CA/CAR cytoprotective effects through anti-inflammatory mechanisms. Overall, the evidence available so far suggests that CA and CAR display anti-inflammatory effects and promote cell survival through regulation of the MAPK pathways. As shown for TAK1, modulation of the earlier-stage (upstream) MAPK pathways by these compounds could also be possible, so further research in this area is required. As a final point, this effect of rosemary diterpenes is in line with their mechanism of action in their anticancer activity via the inhibition of the MAPK and STAT3 (see Section 15.3) pathways [120]. 

### 15.3. Rosemary Diterpenes Modulate the SIRT1/SERT3 Pathways

**Figure 4:**
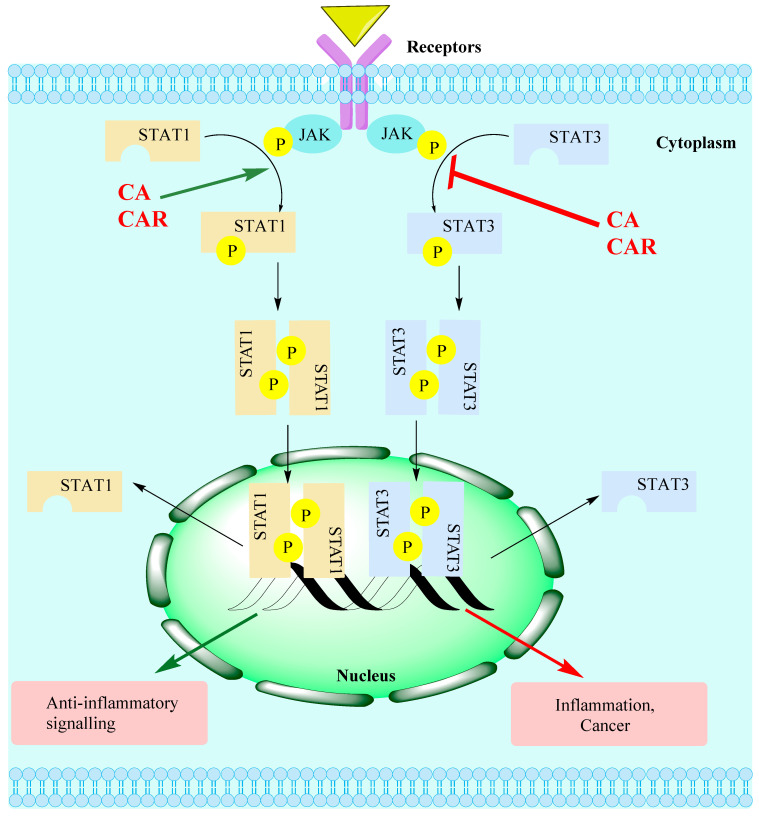
The JAK-STAT signalling pathway targets for CA/CAR. Several external stimuli such as UV radiation, infection, carcinogens, inflammatory mediators and stress can induce the activation of the STAT pathway. Activation of the JAK/STAT pathway associated with receptors’ activation (e.g., by cytokines) leads to the phosphorylation of JAKs. This, in turn, activates STAT monomers through phosphorylation of tyrosine residues, leading to STATs’ dimerisation. Translocation of activated STAT dimers to the nucleus and subsequent DNA binding activate target genes relevant to various physiological/pathological pathways including inflammation. The level of STAT1/3 activation has been shown to be suppressed by rosemary diterpenes, while SIRT1 has been shown to be activated.

The STAT signalling pathway relates to the JAK family protein as an upstream regulator. The STATs (including STAT1, STAT2, STAT3, STAT4, STAT5a, STAT5b and STAT6) control various biological functions, and STAT3 is the most characterised in terms of its role in cell proliferation, survival, differentiation and angiogenesis. When cells are induced by a variety of agents such as cytokines and growth factors, activation of STATs via phosphorylation leads to dimerisation and nuclear translocation, DNA binding and target genes’ activation. Overactivition of STAT3 under inflammatory and cancer conditions has led to suggestions that it is a potential therapeutic target. Evidence along this line came from the fact that inhibition of STAT3 inhibits cancer cells’ proliferation and selectively induces apoptosis in cancer cells. Several forms of oncogenic signalling employ STAT3, and chemotherapeutic resistance has also been associated with overactivation of STAT3 [121,122]. Overall, STAT3 is constitutively expressed in cancer cells (only transiently in normal cells) and is involved in the regulation of genes involved in cancer cells’ survival, invasion, angiogenesis and interaction with immune cells. In the latter case, its role in inflammation has been driving research searching for novel drugs for asthma, inflammatory bowel disease and fibrosis, among others [123]. The phosphorylation of both STAT1 and STAT3 has been shown to be inhibited by CA and CAR through the mechanism depicted in Figure 4. In view of the current level of interest in STATs, further research in this field is needed to assess the true potential of CA and CAR as therapeutic leads through this mechanism of action. Meanwhile, SIRT1 opposes STAT3, and its main role is in the expression of genes that ameliorate stress, apoptosis, aging and inflammation [124]. Several isoforms of the SIRTs have been characterised in recent years but the most studied is SIRT1. As a histone deacetylase enzyme, they are mostly found in the nucleus, and their dysregulation appears to be associated with various disease conditions. Although the complexity of the signalling pathway of SIRT1 as a mechanism for the anti-inflammatory activity of rosemary diterpenes is not yet established, activation of the SIRT1 pathways not only suppresses the production of pro-inflammatory cytokines but also the NLRP3 inflammasome signalling pathway (Section 15.5), as established using the most potent SERT1 activator natural product, resveratrol [125,126,127,128,129,130], and the MAPK pathways of inflammation and neuroprotection [131,132]. SIRT1 also protects cells against oxidative stress by various mechanisms including an increase in the expression antioxidant enzymes. Interestingly, many other natural antioxidants such as quercetin [133,134], berberine [135,136] and curcumin [137,138] have been shown to have cytoprotective, antioxidant and anti-inflammatory effects via upregulation of SIRT1 activity. 

### 15.4. Rosemary Diterpenes Activate the Nrf2/HO-1 Pathways of Cytoprotection

**Figure 5:**
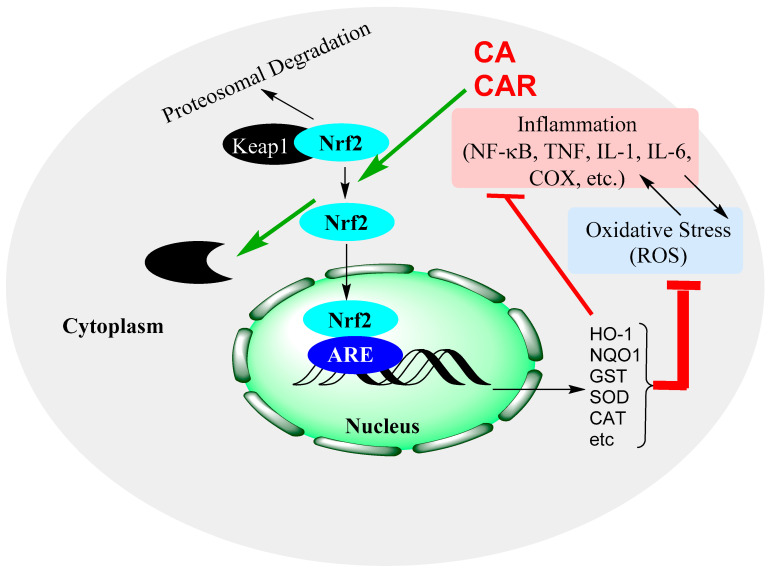
Induction of the Nrf2 signalling pathway and modulation by CA and CAR. Nrf2 activation leads to the expression of antioxidant genes, which, in turn, increase the levels of antioxidant proteins that resolve oxidative stress in cells. The pathway also inhibits inflammatory genes’ activation and has indirect impacts by removing the oxidative stress component of inflammation, oxidative stress crosstalk.

Previous review articles from our laboratories and others showed the therapeutic potential of natural products via upregulation of the Nrf2/HO-1 pathway [139,140,141]. Cellular stress induced by a variety of agents including ROS activates this pathway of cell survival, as outlined for CA and CAR in Figure 5. 

### 15.5. Rosemary Diterpenes Suppress the NLRP3 Inflammasome

The inflammasomes are complexes of large-molecular-weight proteins with functional components such as sensors, adaptors (e.g., apoptosis-associated speck-like protein containing a caspase-recruitment domain or ASC) and pro-caspases. The NLRP3 inflammasome is the best example, which is involved both in the innate immune system and inflammatory signalling in health and disease. In the inflammasome complex of NLRP3, the NLRP3 protein binds to ASC, which, in turn, interacts with pro-caspase-1; in this way, the NLRP3–ASC–pro-caspase-1 complex is formed. This recruitment of NLRP3 leads to the cleaving by caspase-1 of pro-cytokines (e.g., pro-IL-1β) into their mature form, thereby activating inflammatory signalling. In immune cells such as macrophages, various triggers for priming and subsequent activation of NLP3 have been identified. Among the various activators of the NLRP3 inflammasome are ion fluxes such as potassium efflux [142], Ca^2+^ mobilisation from cellular storage sites [143,144], ROS generation and/or mitochondrial dysfunction [145] and lysosomal damage [146]. In terms of the NLRP3 inflammasome’s role in inflammatory diseases, rheumatoid arthritis [147], diabetes [148], cancer [149] and neurodegenerative diseases [150] are just a few to mention. In this emerging role of NLRP3 as a therapeutic target for numerous diseases, CA and CAR have been shown to suppress inflammation activation under various experimental conditions. This is in line with results for other natural products such as apigenin [151], caffeic acid phenethyl ester [152], curcumin [153], resveratrol [154,155] and quercetin [156], among others. The effect could be related to the known effects of lowering the level of ROS and ameliorating mitochondrial dysfunction. 

## 16. Conclusions

Rosemary diterpenes are examples of natural products with phenolic structures that scavenge ROS or remove them indirectly via upregulation of antioxidant defences such as the GSH level and SOD, CAT and GPx activities. They also conduct anti-inflammatory activities by modulating various signalling pathways of inflammation including the NF-κB, MAPK, Nrf2, SIRT and NRLP3 inflammasomes, among others. Through such diverse effects, they downregulate the expression of pro-inflammatory cytokines (e.g., TNF-α, IL-1 and IL-6), adhesion molecules, chemokines and prostaglandins. The therapeutic potential of these diterpenes, as with many natural products, is based on targeting the inflammatory component of the disease through multiple mechanisms. 

## Figures and Tables

**Table 1:** Overview of anti-inflammatory mechanisms of CA and CA based on in vitro studies.

Experimental Model	Compound (Dose)	Main Finding	Reference
RAW 264.7 cells activated by Gram-positive bacteria-derived peptidoglycan, pam3CSK or LPS	CA (5–20 μg/mL)	Inhibits the release of NO, TNF-α and PGE₂; inhibits NF-κB activation and the phosphorylation of Syk/Src, PI3K, Akt, IκBα, IKK and IκBα.	Oh et al. [19]
RAW 264.7 cells activated by LPS	CA and CAR (10 μM)	Suppresses MMP-9 and MCP-1 release.	Chae et al. [20]
RAW 264.7 cells activated by LPS	CA and CAR (12.5–50 μg/mL)	Suppresses NO production.	Mengoni et al. [21]
RAW 264.7 cells activated by LPS	CA (2.5–20 μM)	Inhibits NO, TNF-α and COX-2 expression; suppresses the transcription of inflammatory genes (*Nos2*, *Tnfα*, *Cox*2 and *Mcp*1); inhibits IKKβ/IκB-α/NF-κB, MAPKs (ERK, JNK and p38) and FoxO1/3 signalling pathways.	Wang et al. [22]
RAW 264.7 cells activated by LPS	CAR (IC_50_ 9.4 μM)	Inhibits NO production and iNOS expression (mRNA and protein); inhibits NF-κB translocation and DNA binding activity; inhibits IKK activity and degradation of IκBα; inhibits MAPK (p38 and p44/42) activation.	Lo et al. [23]
RAW 264.7 cells activated by LPS	CAR (1, 2 and 5 μM)	Inhibits NO and expression of iNOS and COX-2; inhibits STAT3 phosphorylation and DNA binding activity.	Lee et al. [24]
RAW 264.7 cells activated by LPS	CA and CAR (5–15 μM)	Inhibits NO and PGE₂, cytokine (IL-1α and IL-6) and chemokine (CCL5/RANTES, CXCL10/IP-10) production, along with gene expression of iNOS; suppresses nuclear translocation of NF-κBp65.	Schwager et al. [25]
Primary mouse bone-marrow-derived macrophages (BMDMs) simulated by LPS	CAR (2.5–40 µM)	Inhibits NLRP3 inflammasome activation and HSP90; inhibits pro-inflammatory cytokine (pro-IL-1β, TNF-α and IL-6) expression.	Shi et al. [26]
Human whole-blood simulated by LPS	CA and CAR (IC_50_ 1.9–3.5 μg/mL)	Inhibits the activity of microsomal PGE_2_ synthase (mPGES)-1.	Bauer et al. [27] Maione et al. [28]
Mouse bone-marrow-derived mast cells stimulated by anti-TNP IgE	CA (15 and 50 μM)	Inhibits ROS generation, Ca^2+^ mobilisation and degranulation; suppresses protein and gene expression of pro-inflammatory cytokines (IL-6, IL-13 and TNF) and chemokines (CCL2, CCL3 and CCL9); reduces phosphorylation of IKK and IκBα, Syk (Tyr352 and 525/526), TAK1 (Ser412) and Akt; decreases the level of *NFKB2* mRNA and genes (*c-jun*, *Egr1* and *Egr2*).	Crozier et al. [29]
BV2 mouse microglial cells stimulated by LPS and INF-γ	CA and CAR (5 μM)	Inhibits NO and TNF-α, and PGE_2_ production; induces HO-1 expression.	Foresti et al. [30]
IL-1β- or TNF-α-stimulated human periodontal ligament cells	CA (3.125–50 µM)	Suppresses the release of IL-6 and chemokines’ (CXCL10, CCL2 and CCL20) production; inhibits JNK, NF-κB and STAT3.	Hosokawa et al., 2020 [31]
Human oral epithelial cell line (TR146 cells) stimulated by IL-27	CA (3.125–50 µM)	Suppresses chemokine (CXCL9, CXCL10 and CXCL11) production; inhibits the phosphorylation of STAT1, STAT3 and Akt.	Hosokawa et al., 2019 [32]
Bone marrow cells and osteoblasts stimulated by M-CSF	CA (10 or 20 μM)	Inhibits ROS production while augmenting SOD and GPx activity; inhibits the RANKL-mediated activation of NF-κB and MAPKs (JNK and p38) and expression of cytokines (TNF-α, IL-1β and IL-18) and COX2.	Liu et al. [33]
Chondrosarcoma cell line SW1353 and primary human chondrocytes stimulated by IL-1β	CA, and CAR (5–15 µM)	Inhibits catabolic genes such as MMP-13 and ADAMTS-4 and nuclear translocation of NF-κBp65.	Schwager et al. [25]
Human neutrophils stimulated by fMLF, MMK1 or PMA	CA (1–10 μM)	Suppresses the expression of integrin adhesion molecules (CD11b) and adhesion of neutrophils to endothelial (bEND 3) cells; inhibits the phosphorylation of MAPKs (ERK, JNK and p38).	Tsai et al. [34]
Human lung NCI-H1975 cells for H_2_O_2_-induced cell death; excised-lung organ culture ischemia model	CAR (3 μM)	Cytoprotection via upregulation of HO-1.	Kawamura et al. [35]
Keratinocyte HaCaT cells stimulated with SLS and RA	CA (5–20 μg/mL)	Suppresses the production of IL-6, IL-8 and MCP-1.	Oh et al. [19]
SH-SY5Y cells exposed to paraquat	CA (1 μM)	Inhibits NF-κB transcription and IL-1β, TNF-α and COX-2 expression; effect mediated via activation of the Nrf2 and HO-1 signalling pathway.	de Oliveira et al. [36]
PC12 cells subjected to serum starvation	CAR (10 µM)	Cytoprotective effect via activation of the HO-1 and Nrf2 pathway.	Martin et al. [37]
6-OHDA-induced neuronal (SH-SY5Y) cell death	CA (1 µM)	Cytoprotective effect through inhibition of the MAPK pathway; inhibition of phosphorylation of JNK and p38.	Wu et al. [38]
PC12 cells; hypoxia-induced neuronal cell injury model	CA (1 μM)	Improves cell viability; suppresses ROS generation and lipid peroxidation; PGE_2_ (also COX-2 activation) NO and pro-inflammatory cytokines (IL-1 and IL-6) production; and ERK, JNK and p38 MAPK activation.	Hou et al. [39]
SH-SY5Y	CA (30 µM)	Inhibits Aβ (1-40 and 1-42) production by activating α-secretase, TACE.	Meng et al. [40]
U373MG human astrocytoma cells	CA (50 μM)	Inhibits Aβ peptides (1-40, 1-42 and 1-43) by increasing mRNA expression of α-secretase (*TACE*).	Yoshida et al. [41]
3T3-L1 adipocytes stimulated by TNF-α	CA (1–20 µM)	Inhibits mRNA expression of inflammatory genes (*IL-6* and *MCP-1*), the activation of ERK and JNK, the phosphorylation of IκB and IKK, the nuclear translocation of p65 and the DNA-binding activity of NF-κB and AP-1.	Tsai et al. [42]
Rat cardiomyocytes (H9C2 cells); DOX-induced cardiotoxicity	CA (2.4–10 µM)	Suppresses production of ROS and NO and activation or phosphorylation of p38 and JNK; inhibits NF–κB (p65) activation; upregulates Nrf2 and HO-1 levels.	Manna et al. [43]
H9C2 cells; DOX-induced cardiotoxicity	CA (5–20 μM)	Suppresses the level of pro-inflammatory cytokines (TNF-α, IL-6, IL-1β and IL-18) and COX-2; inhibits NF-κB activation.	Zhang et al. [44]
H9C2 cells stimulated by LPS	CAR (5–20 μM)	Inhibits NF-*κ*B activation and cytokine (TNF-*α*, IL-1*β*, IL-6) and COX-2 (as well as PGE_2_) expression; possible direct interaction with IKK*β* (in silico study).	Baradaran Rahimi et al. [45]
HepG2 cells exposed to ethanol (100 mM)	CA (10 µM)	Inhibits oxidative stress, inflammation and cell death; effect mediated by activation of SIRT1 (see also in vivo effect).	Gao et al. [46]
LPS-treated hepatic stellate cells from mice	CA nanoparticles	Deactivates phosphorylated IKKα, IκBα and NF-κB; decreases TNF-α, IL-1β and IL-18 expression; suppresses ROS production while increasing SOD1, SOD2, HO-1 and Nrf-2 levels.	Li et al. [47]
3T3-L1 adipocytes stimulated by LPS	CA (up to 20 µM)	Suppresses TNF-α, IL-6 and MCP-1 mRNA levels; downregulates NF-κB and ERK.	Park and Mun, [48]
Human retinal endothelial cells challenged by high glucose	CAR (2.5–20 µM)	Upregulates the expression and activity of Nrf2, HO-1 and ERK1/2; suppresses ROS production and apoptosis.	D’Agata et al. [49]
Human lung microvascular endothelial cells (HMVEC-L) challenged by t-BHP	CAR (10 µM)	Increases the expression of Nrf2 and HO-1 while it also interrupts the Nrf2-Keap1 protein−protein interaction; inhibits cell death.	Li et al. [50]
HCT-116 cells challenged by thapsigargin	CAR (10 µM)	Ameliorates the induced endoplasmic reticulum stress; suppresses the expression of pro-inflammatory mediators (TNF-α, IL-6, IFN-γ, CXCL10).	Xu et al. [51]

Abbreviations: 6-OHDA, 6-hydroxydopamine; Akt, protein kinase B; CCL5/RANTES, CC chemokine ligand; CXCL10/IP-10, C-X-C motif chemokine ligand 10; COX-2, cyclooxygenase-2; DOX, doxorubicin, ERK, extracellular signal-regulated kinase ½; fMLF, N-formyl-L-methionyl-L-leucyl-*L*-phenylalanine; GPx, glutathione peroxidase; IL, interleukin; HO-1, haem oxygenase; iNOS, inducible nitric oxide synthase; INF-γ, interferon-γ; IκBα, nuclear factor of kappa light polypeptide gene enhancer in B-cells inhibitor-α; IKK, IκB kinase complex; JNK, c-Jun N-terminal kinases; MAPKs, mitogen-activated protein kinases; M-CSF, macrophage colony-stimulating factor; MCP-1, *monocyte chemoattractant protein*-*1*; MMK1, synthetic peptide agonist for formyl peptide receptor-2; MMP-9, matrix metallopeptidase 9; mPGES, microsomal prostaglandin E synthase; NF-κB, nuclear factor-κB; NO, nitric oxide; Nrf2, nuclear factor erythroid 2-related factor 2; PGE2, prostaglandin E2; PMA, phorbol-12-myristate-13-acetate; RA, retinoic acid; RANKL, receptor activator for nuclear factor-κB ligand; ROS, reactive oxygen species; SLS, sodium lauryl sulphate; Src, proto-oncogene tyrosine-protein kinase; STAT3, signal transducer and activator of transcription-3; Syk, spleen-associated tyrosine kinase; TNF-α, tumour necrosis factor-α.

**Table 2:** Overview of anti-inflammatory mechanisms of CA and CA based on in vivo studies.

Experimental Model	Compound (Dose)	Main Finding	Reference
LPS-induced septic shock in mice	CAR (20 or 40 mg/kg, i.p.)	Prevents NLRP3 inflammasome activation; downregulates the serum levels of IL-1β and TNF-α.	Shi et al. [26]
Methionine- and choline-deficient (MCD) diet-fed mouse NASH model	CAR (20 or 40 mg/kg, i.p.)	Suppresses liver injury, fibrosis, NLRP3 inflammasome activation, IL-1β, TNF-α and profibrotic marker alpha-smooth muscle actin (α-SMA).	Shi et al. [26]
Adjuvant arthritis model in rats	Methotrexate (0.3 mg/kg) in combination with CA (100 mg/kg, p.o.)	Suppresses hind paw swelling, the levels of IL-17A, MMP-9 and MCP-1 in plasma, and GGT activity in the joint; increases mRNA expression levels of HO-1 and CAT; suppresses IL-1β level in the liver.	Chrastina et al. [52]
Collagen-induced arthritis-db/db mice model of rheumatoid arthritis	CA (30 and 60 mg/kg, i.p.)	Improves bone loss coupled with antidiabetic effects (e.g., OGTT and ITT).	Xia et al. [53]
Type II collagen-induced arthritis model in mice	CAR (40 mg/kg, p.o.) or rosmanol (40 mg/kg/d, p.o.)	Alleviates swelling, redness and synovitis; decreases the arthritis index score and the serum level of pro-inflammatory cytokines (IL-6, MCP-1 and TNF-α); blocks NF-κB and MAPK (JNK and p38 MAPK) pathways; better result in drug combination with rosmanol.	Li et al. [54]
ARDS in mice induced by LPS	CA (5 or 10 mg/kg, i.v.)	Improves inflammatory status (histology); reduces MPO activities, neutrophil infiltration and lipid peroxidation.	Tsai et al. [34]
LPS-induced acute lung injury (ALI) experimental model in mice	CA (10, 20 and 40 mg/kg, i.p.)	In addition to histological improvement, reduces the production (mRNA and protein) of IL-1β, IL-6, TNF-α, TLR4 and NF-κB expression and NF-κB phosphorylation in lung tissues.	Li et al. [55]
Bleomycin-induced lung damage in rats	CAR (10, 20 and 40 mg/kg, p.o.)	Reduces oxidative markers (MDA, NO, protein carbonyl), proinflammatory cytokines (TNF-α and IL-6 levels) and MPO activity in the lungs; increases GSH content and activities of CAT, GPx and SOD; reduces lung fibrosis.	Kalantar et al. [56]
Ovalbumin-induced allergic asthma in mice	CAR (5 mg/kg, i.p.)	Reduces eosinophils in the bronchoalveolar lavage fluids, and pro-inflammatory cytokines’ production (IL-4 and IL-13) in the bronchoalveolar lavage fluids and the lungs.	Lee and Im [57]
PMA-induced ear inflammation in mice	CA and CAR-EC_50_ values for reduction of oedema of 10.20 μg/cm^2^ and 10.70 μg/cm^2^, respectively	Reduces oedema, ulceration, leucocyte infiltration and expression levels of IL-1β, TNF-α and COX-2.	Mengoni et al. [21]
Carrageenan-induced oedema model in mice	CAR (1–10 mg/ kg, i.p.)	Reduces oedema; decreases MPO, NO and IL-17A; increases the level of anti-inflammatory cytokine, IL-10.	da Rosa et al. [58]
Atopic dermatitis in mice induced by 5% phthalic anhydride	CAR (0.05 µg/cm^2^)	Inhibits the expression of iNOS and COX-2 in skin tissue; inhibits STAT3 in skin tissue; reduces the serum levels of TNF-α, IL-1β and IgE.	Lee et al., 2017 [24]
UVB-induced skin inflammation in mice	CAR (0.05 µg/cm^2^)	Reduces erythema, epidermal thickness and serum levels of IgE and IL-1β; suppresses iNOS and COX-2; decreases activation of STAT3 and JAK.	Yeo et al. [59]
Carrageenan-induced oedema model in mice	CA (30 or 100 µg per paw)	Reduces oedema and levels of microsomal prostaglandin E synthase-1 (mPGES-1) and 5-LO-derived products.	Maione et al. [28]
6-OHDA model of PD in rats	CA (20 mg/kg, p.o.)	Improves behavioural changes along with LPO, GSH and SOD.	Wu et al. [38]
Chlorpyrifos-induced neuronal damage in mice	CA (30 and 60 mg/kg p.o.)	Suppresses the serum level of pro-inflammatory cytokines (IL-1β, IL-6 and TNF-α) in cerebral and ocular tissues, reverses the decrease in AChE and antioxidant markers (GSH, GPx, SOD and CAT) and reduces pro-oxidant (MDA and NO) markers.	AlKahtane et al. [60]
Mild TBI in mice	CA (1 mg/kg, i.p.)	Improves motor and cognitive dysfunction, activates Nrf2 and suppresses NF-κB.	Maynard et al. [61]
Spinal cord injury in rats	CAR (5 mg/kg, i.p.)	Activates Nrf2; reduces ROS generation, LPO content, protein carbonyl and sulfhydryl levels; increases antioxidant status (SOD, CAT GPx, GSH, GSH-*S*-transferase); inhibits NF-κB and COX-2 expression; reverses the reduction in phosphor-Akt.	Wang et al. [62]
Traumatic brain injury in mice	CA (0.3, 1.0 or 3.0 mg/kg, i.p.)	Activates the Nrf2–ARE pathways; improves mitochondrial respiratory dysfunction, lipid peroxidation and protein nitration in brain tissues.	Miller et al. [63]
Subarachnoid haemorrhage brain injury model in rats	CA (3 mg/kg, i.p.)	Increases SIRT1, MnSOD and Bcl-2 in addition to improving brain oedema and neuronal structure and function.	Teng et al. [64]
APP/PS1 mouse model of AD	CA (10 or 30 mg/kg, p.o)	Reduces Aβ deposition, cognitive decline and levels of pro-inflammatory cytokine (IL-1β, TNFα and IL-6) production; inhibits Aβ secretion and interaction between CEBPβ and NFκB p65.	Yi-Bin et al. [65]
Experimental autoimmune encephalomyelitis in mice	CAR (50 mg/kg, i.p.)	Reduces demyelination and inhibits Th17 cell differentiation and STAT3 phosphorylation; blocks translocation of NF-κB; switches macrophage/microglia to non-inflammatory phenotype.	Li et al. [66]
STZ-induced diabetic rats	CAR (1, 5, 10 mg/kg/day, i.p. for 4 weeks)	Suppresses serum levels of glucose, IL-6, TNF-α, MDA, TG, TC, LDL-C, GST, SOD, CAT and HDL-C in a dose-dependent manner.	Samarghandian et al. [67]
STZ-induced diabetes in rats	CA (30 mg/kg)	Reduces glucose level in diabetic rats; reduces MDA and glycated end products, tissue damage and inflammation score; reverses change in the gut microbiota population.	Ou et al. [68]
STZ-induced diabetic mice db/db mice	CA (15 or 30 mg/kg, i.g.)	Nephroprotective effect coupled with activation of Nrf2 and inhibition of NF-κB.	Xie et al. [69]
Ischaemia/reperfusion model in diabetic mice	CA (50 mg/kg, p.o.)	Suppresses ROS and pro-inflammatory cytokine (IL-6 and TNF-α) production.	Hu et al. [70]
DOX-induced cardiotoxicity in rats	CA (10 mg/kg, p.o.)	Decreases the levels of ROS, NO, phospho-p38, phospho-JNK1 proteins and NF–κB (p65); reverses the downregulation of Nrf2 in the nucleus and HO-1 in the cardiomyocytes.	Manna et al. [43]
DOX-induced cardiotoxicity mice	CA or Carvedilol (5 mg/kg, p.o.)	Ameliorates cardiac injury and suppresses the levels of pro-inflammatory cytokines (TNF-α, IL-6, IL-1β and IL-18) and COX-2, and NF-κB; reverses the reduced antioxidant level (GSH) or activity (SOD, CAT and NQO-1) and the increased oxidative stress (MDA level); increases Nrf2 in heart tissue; drug combination offers better result.	Zhang et al. [44]
Chronic alcoholic liver injury model in rats	(15 or 30 mg/kg, i.g.)	Activates SIRT1 and increases MnSOD; suppresses NF-κB and serum level of TNF-α.	Gao et al. [46]
Ischemia/reperfusion model of liver damage in rats	CA (10 and 20 mg/kg, i.p.)	Normalises the levels of SOD, CAT and GSH and GPx) and the NF-κB signalling pathway of pro-inflammatory cytokine (TNF-α and IL-1β) expression.	Li et al. [47]
HFD-induced NAFLD model in mice	CA (15 mg/kg, p.o.)	Improves glucose and insulin tolerance; suppresses the serum and hepatic levels of IL-1β, IL-18, TNF-α, IL-2, IL-4, IL-6, IL-12 and IFN-γ; reverses the low-level MARCKS under diabetes; ameliorates the diabetes-associated activation of PI3K/Akt, NLRP3/NF-κB and SREBP-1c signalling pathway.	Song et al. [71]
LPS-induced liver injury in rats	CA (30 or 60 mg/kg, p.o.)	Ameliorates liver damage (histology and biochemical markers) and suppresses inflammatory cells’ infiltration and the serum level of pro-inflammatory cytokines (TNF-α and IL-6); increases antioxidant levels (SOD, GSH and GPx) in serum and liver.	Xiang et al. [72]
LPS-induced liver injury in mice	CA (40 mg/kg, i.p.)	Inhibits the expression of pro-inflammatory cytokines (IL-1β, IL-6, TNF-α and MCP-1, mRNA and protein), NOX4 (mRNA and protein) immune cell (neutrophil) infiltration and NF-κB activation; increases GSH, CAT and MnSOD.	Kim et al. [73]
Renal ischemia-reperfusion injury in rats	CAR (3 mg/kg, i.v.)	Inhibits apoptotic tubular cell death and activation of the p38 pathway.	Zheng et al., 2018 [74]
NASH model in mice	CAR (20 or 40 mg/kg, i.p.)	Suppresses NLRP3 inflammasome activity via direct effect on heat-shock protein 90 (HSP90).	Shi et al. [26]
HFD-induced mouse obesity and metabolic syndrome model	CA (10 or 20 mg/kg, p.o.)	Downregulates the levels of pro-inflammatory cytokines (IL-1β, IL-6 and TNF-α) in serum and brain tissues, and the NF-κB signalling pathway.	Liu et al. [75]
Dextran sulphate sodium (DSS) experimental model of colitis mice	CAR (50 mg/kg i.p.)	Reduces inflammatory cell infiltration and pro-inflammatory cytokine (TNF-α, IL-1β, IL-6 and IFN-γ) expression.	Xu et al. [51]

Abbreviations: See also Table 1 legend. Aβ, β-amyloid; AChE, acetylcholinesterase; AD, Alzheimer’s disease; ARDS, acute respiratory distress syndrome; CAT, catalase; GGT, gamma-glutamyltransferase; HDL-C, high-density lipoprotein cholesterol; HFD, high-fat-diet; i.g., intragastric; i.p., intraperitoneal; i.v., intravenous; IgE, immunoglobulin E; ITT, insulin tolerance test; JAK, Janus kinase; LDL-C, low-density lipoprotein cholesterol; MARCKS, myristoylated alanine-rich C-kinase substrate; MDA, malondialdehyde; MPO, myeloperoxidase; OGTT, oral glucose tolerance test; NAFLD, non-alcoholic fatty liver disease; NASH, non-alcoholic steatohepatitis; NOX4, nicotinamide adenine dinucleotide phosphate oxidase 4; NQO-1, NAD(P)H quinone dehydrogenase; 1PD, Parkinson’s disease; phosphor, phosphorylated; PI3K, phosphoinositide 3-kinases; p.o., oral administration; SREBP-1c, sterol regulatory element-binding protein-1c; TBI, traumatic brain injury; TC, total cholesterol; TG, triglycerides; TLR4, Toll-like receptor.

## Data Availability

Not applicable.
